# Integrating chronic inflammation and hypoxia: the potential role of HIF-1α in tumor behavior and therapy response in high-grade serous ovarian cancer

**DOI:** 10.3389/fimmu.2026.1757708

**Published:** 2026-03-09

**Authors:** Sara Polajžer, David Lukanović, Erik Škof, Borut Kobal, Katarina Černe

**Affiliations:** 1 Institute of Pharmacology and Experimental Toxicology, Faculty of Medicine, University of Ljubljana, Ljubljana, Slovenia; 2 Department of Gynecology, Division of Gynecology and Obstetrics, Ljubljana University Medical Centre, Ljubljana, Slovenia; 3 Department of Gynecology and Obstetrics, Faculty of Medicine, University of Ljubljana, Ljubljana, Slovenia; 4 Department of Medical Oncology, Institute of Oncology Ljubljana, Ljubljana, Slovenia; 5 Faculty of Medicine, University of Ljubljana, Ljubljana, Slovenia

**Keywords:** body fluids, chronic inflammation, HIF-1α, high-grade serous ovarian carcinoma, neoadjuvant chemotherapy, tumor tissue

## Abstract

High-grade serous ovarian carcinoma (HGSOC) is marked by late diagnosis and chemoresistance, partly driven by chronic inflammation and hypoxia in the tumor microenvironment. Hypoxia-inducible factor 1-alpha (HIF-1α) is a key regulator of these processes; however, its spatial distribution, interaction with inflammation, and effect on chemotherapy response in HGSOC remain unclear. This retrospective study included 28 advanced HGSOC patients treated with neoadjuvant chemotherapy (NACT). Samples were collected at primary surgery (PS) (ovarian and peritoneal tissue, plasma, ascites) and post-NACT at interval debulking surgery (IDS) (omentum, peritoneal tissue, plasma). HIF-1α mRNA expression varied by site, with higher levels in omental and peritoneal tissues compared to ovarian tissue. Plasma and ascites concentrations were significantly correlated, although the mean ascites concentration was lower. Elevated HIF-1α concentration in ascites and plasma at baseline correlated with ESR (erythrocyte sedimentation rate) >30 mm/h, which was also correlated with *BRCA* mutation status. No correlation was found between HIF-1α and CRP (C-reactive protein) levels. Higher HIF-1α concentrations in ascites and plasma were linked to poor chemotherapy response (CRS1) at IDS. No significant changes in plasma HIF-1α, ESR, or peritoneal HIF-1α mRNA expression were observed before and after chemotherapy. Increased peritoneal HIF-1α at baseline showed a trend toward shorter progression-free survival. These findings suggest that HIF-1α may reflect hypoxia-inflammation crosstalk associated with chemoresistance and progression in HGSOC. The hypoxic-inflammatory microenvironment appears to persist despite chemotherapy and could contribute to ongoing disease activity. However, these observations require validation in independent cohorts before any prognostic or predictive implications can be considered.

## 1 Introduction

Chronic inflammation is increasingly recognized as a central driver of cancer progression, shaping the tumor microenvironment to promote immune dysregulation, angiogenesis, extracellular matrix remodeling, and metastatic spread (1, 2). In high-grade serous ovarian carcinoma (HGSOC), the most common and lethal subtype of epithelial ovarian cancer, sustained inflammatory signaling contributes to chemoresistance and poor long-term survival, particularly in patients diagnosed at advanced stages with widespread peritoneal dissemination (3–5).

Hypoxia-inducible factor 1-alpha (HIF-1α) is a key mediator connecting hypoxia to chronic inflammatory signaling (6). Stabilized under low-oxygen conditions, HIF-1α drives local and systemic immune responses, including immune cell recruitment and pro-inflammatory cytokine production, while also enabling metabolic adaptation and survival of tumor cells (6–9). These processes establish a persistent pro-tumorigenic microenvironment and contribute to chemoresistance through enhanced DNA repair, apoptosis inhibition, and metabolic reprogramming (10). Despite growing recognition of the role of hypoxia in HGSOC, it remains unclear how HIF-1α mRNA expression is distributed across the major anatomical and fluid compartments that define the disease—namely the ovary, omentum, peritoneum, ascites, and plasma. Similarly, the extent to which HIF-1α in tumor tissues and body fluids relates to systemic inflammatory activation, including erythrocyte sedimentation rate (ESR) and C-reactive protein (CRP), is not well established. Furthermore, although hypoxia is increasingly implicated in chemoresistance, the prognostic and predictive relevance of HIF-1α regarding chemotherapy response score (CRS), CA-125 ELIMination Rate Constant K (KELIM), platinum-free interval (PFI), and progression-free survival (PFS) remains insufficiently defined in HGSOC.

To address these gaps, we conducted a comprehensive, multi-compartment analysis of HIF-1α across tumor tissues, ascitic fluid, and plasma at both primary surgery (PS) and interval debulking surgery (IDS) in patients referred to neoadjuvant chemotherapy (NACT). This design allowed us to interrogate spatially distinct hypoxic niches and evaluate their stability or modulation in response to NACT. Our primary aim was to elucidate how local and systemic HIF-1α mRNA expression contributes to the chronic inflammatory and therapy-resistant tumor microenvironment characteristic of HGSOC. We further examined the associations between HIF-1α and systemic inflammation, chemotherapy response and disease progression.

## 2 Materials and methods

### 2.1 Study design

This retrospective cohort study included patients diagnosed with advanced-stage (FIGO stage III or IV) primary HGSOC between March 2021 and October 2025 at the Department for Gynecology, University Medical Centre Ljubljana. The study was conducted within the framework of the original study protocol, using the same patient cohort, inclusion criteria. methodological procedures, and study objective. The study was registered at ClinicalTrials.gov (reference number NCT05490407). Prior to participation, all patients provided written informed consent. The study protocol was approved by the National Medical Ethics Committee of the Republic of Slovenia (0120-316/2019/3), and amended approval was obtained to allow additional biomarker analyses (0120-465/2025-2711-3).

Eligible patients had histologically confirmed primary HGSOC, FIGO stage III or IV disease, presence of ascites at diagnosis, no concurrent malignancies, and were referred NACT. Forty-five women with suspected ovarian cancer were initially evaluated and underwent diagnostic laparoscopy or laparotomy as part of the primary surgical procedure (PS). Based on intraoperative findings and clinical assessment, 28 patients were referred for NACT; five did not complete the planned treatment protocol and were excluded from final analyses.

NACT consisted of 3–6 cycles of platinum-based chemotherapy prior to interval debulking surgery (IDS), followed by 0–3 additional cycles postoperatively. Standard regimens included carboplatin combined with paclitaxel, with carboplatin monotherapy administered when combination therapy was contraindicated. Maintenance therapy with Poly (ADP-ribose) polymerase (PARP) inhibitors (niraparib or olaparib) was introduced when indicated. Bevacizumab, an anti-VEGF monoclonal antibody, was incorporated into the chemotherapy regimen when clinically appropriate.

Plasma, ascites, ovarian, and peritoneal tissue samples were collected at PS, while blood, peritoneal, and omental tissue samples were obtained at IDS. Ascites was not routinely available at IDS due to its absence following successful NACT (11). Tumor response was assessed using the CRS (12, 13), KELIM (14, 15), and PFI (16).

A detailed study design and schematic representation (**Supplementary Figure 1**.), together with the methodology for KELIM calculation, CRS categorization, and PFI definition, is provided in **Supplementary Material**.

### 2.2 Sample collection

Venous blood samples for HIF-1α analysis were collected during the patients’ hospital admission for preoperative preparation before both PS and IDS. A total of 6 mL of peripheral blood was drawn into BD Vacutainer^®^ EDTA tubes (BD, Columbus, NV, USA) and kept on ice for no longer than 30 min prior to centrifugation at 1000 × g for 15 min at 4 °C. At the start of surgery, immediately upon entering the abdominal cavity, 50 mL of ascitic fluid was aspirated using a sterile syringe, transferred into a conical tube, and maintained on ice until centrifugation under the same conditions (1000 × g, 15 min, 4°C). Plasma and ascites supernatants were aliquoted and stored at −80°C until further analysis. As part of routine surgical management, the primary tumor specimen was submitted for histopathological evaluation. For HIF-1α detection, at least 1 cm³ of tumor tissue was collected, flash-frozen in liquid nitrogen, and subsequently stored at −80°C.

### 2.3 ELISA analysis of HIF-1α

HIF-1α concentrations in ascites and plasma were quantified using a Human HIF-1α ELISA kit (CSB-E12112h, CUSABIO, Wuhan, China) following the manufacturer’s instructions. Samples weren’t diluted prior to analysis. Absorbance was measured at 450 nm and at 540 nm using a Synergy HT microplate reader (BioTek, Shorline, WA, USA). Readings at 540 nm were subtracted from the 450 nm values to correct for plate-related optical imperfections. A seven-point standard curve (62.5–4000 pg/ml) was generated in CurveExpert 1.4, using a 4th-degree polynomial fit. The kit’s lower limit of detection was 15.6 pg/ml, and all samples were measured in triplicates.

### 2.4 Isolation of RNA, reverse transcription and qPCR of HIF-1α

RNA was extracted from tissue and cell samples using the peqGOLD Total RNA Kit (VWR International GmbH, Vienna, Austria), and cDNA was synthesized with the High-Capacity cDNA Reverse Transcription Kit (Thermo Fisher Scientific, Waltham, MA, USA), following the manufacturers’ instructions. qPCR analyses were performed using HOT FIREPol EvaGreen qPCR Supermix, 5× (Solis BioDyne, Tartu, Estonia) on a LightCycler 480 instrument (Roche Diagnostics, Basel/Rotkreuz, Switzerland). Primer sequences used were HIF-1α forward 5′-TATGAGCCAGAAGAACTTTTAGGC-3′ and reverse 5′-CACCTCTTTTGGCAAGCATCCTG-3′; ACTB forward 5′-CACCATTGGCAATGAGCGGTTC-3′ and reverse 5′-AGGTCTTTGCGGATGTCCACGT-3′; PPIA forward 5′-GGCAAATGCTGGACCCAACACA-3′ and reverse 5′-TGCTGGTCTTGCCATTCCTGGA-3′. Cycling conditions consisted of 95 °C for 12 min, followed by 40 cycles of 95°C for 15 s and 60 °C for 30 s, and a melt-curve analysis. Melt-curve profiles were consistent across all reactions, confirming specificity, and no-template controls (NTCs) were included in each run to exclude contamination. All samples were diluted to 10 ng/µL before amplification and quantified in duplicates. Standard curves were generated from cDNA dilution series, and PCR efficiency was calculated for every reaction, yielding values close to 100%. Absolute quantification was performed using the second-derivative maximum method (LightCycler 480 Software v1.5). *HIF-1α* mRNA expression was normalized to the housekeeping genes *ACTB* and *PPIA*, and relative mRNA expression of *HIF-1α* was calculated using the equation 2^-ΔCt^, where ΔCt= Ct*_HIF-1α_*- geomean (Ct*_ACTB_*,Ct*_PPIA_*), Ct is the cycle threshold.

### 2.5 Statistical analysis

All statistical analyses were performed using R Statistical Software, version 4.5.2 (R Core Team 2025. R Foundation for Statistical Computing, Vienna, Austria). Continuous variables were described using the mean with interquartile range (25–75%). Categorical variables were described using frequencies. The normality of distribution was tested with the Shapiro-Wilk test. The parametric t-test and nonparametric Wilcoxon test compared the distribution of continuous variables among different patient groups. Pearson’s correlation and Spearman’s correlation coefficients were used to assess correlations between continuous and categorical variables. No formal correction for multiple testing was applied due to the limited sample size and exploratory design of the study. Given the number of comparisons made, some statistically significant findings may represent chance associations and should therefore be interpreted with caution.

Receiver operating characteristic (ROC) analyses were performed to assess the prognostic utility of HIF-1α mRNA expression, and the area under the curve (AUC) was calculated. Optimal cut-off values for HIF-1α mRNA expression were determined using the Youden index. Based on ROC analysis and the distribution of progression-free survival (PFS) times within the cohort, a clinically meaningful threshold of 30 months was selected to distinguish early from late progression. Proportional hazards Cox regression models were used to evaluate the association between HIF-1α mRNA expression, PFS and PFI. PFS was defined as the interval from primary surgery to radiologic or clinical evidence of disease progression. Kaplan–Meier survival curves were generated to compare survival outcomes between patients stratified by HIF-1α mRNA expression levels and by PFS < 30 versus ≥ 30 months, and differences between survival curves were assessed using the log-rank test. All the statistical tests were two-sided, with the significance level set at 0.05.

## 3 Results

### 3.1 Study and patient characteristics

The overall study duration was 55.0 months. The mean follow-up for all patients was 29.0 months, with a median of 32.5 months (minimum 1.9 months and maximum 49 months). At the time of analysis, 18 of the 28 patients had died, while 6 patients remained free of detectable disease progression. The 30-month PFS cutoff was selected based on the distribution of progression events within the cohort and the overall follow-up duration, allowing discrimination between early and later disease progression in a clinically meaningful manner. This cutoff was therefore chosen primarily based on the internal distribution of events within our cohort rather than on a predefined or universally established clinical guideline threshold. For some sample types, the number of collected samples is lower than the number of patients, as tissue acquisition was not feasible in certain surgeries. One ovary sample was excluded from the analysis as sampling-related damage resulted in unreliable data. The summarized patient data is presented in **Table 1**.

**Table 1:** Summary of patient data.

Variable	Category	n	%	Mean	SD
mRNA expression of HIF1α in ovaries at PS (2^-ΔCt^)	Numerical variable	26	92.9	0.0055	0.006
mRNA expression of HIF1α in peritoneum at PS (2^-ΔCt^)	Numerical variable	23	82.1	0.0151	0.0114
mRNA expression of HIF1α in omentum at IDS (2^-ΔCt^)	Numerical variable	21	75.0	0.0134	0.007
mRNA expression of HIF1α in peritoneum at IDS (2^-ΔCt^)	Numerical variable	20	71.4	0.0113	0.007
Concentration of HIF1α in ascites at PS (pg/ml)	Numerical variable	28	100.0	443.92	443.44
Concentration of HIF1α in plasma at PS (pg/ml)	Numerical variable	28	100.0	1046.59	763.36
Concentration of HIF1α in plasma at IDS (pg/ml)	Numerical variable	20	71.4	1013.22	694.98
Age (years)	Numerical variable	28	100	63	8.82
KELIM score	< 1	14	50.0	/	/
	≥ 1	9	32.1	/	/
CRS	1-minimal	11	39.2	/	/
	2-partial	7	25.0	/	/
	3-complete/significant	5	17.9	/	/
ESR at PS (mm/hr)	≤30	5	17.9	19.40	7.27
	>30	23	82.1	62.68	21.39
ESR at IDS (mm/hr)	≤30	3	10.71	20.00	8.12
	>30	20	71.43	56.21	25.85
CRP at PS (mg/l)	>5	28	100.0	65.39	53.90
CRP at IDS (mg/l)	>5	2	7.14	66.5	23.33
CA-125 at PS (kU/l)	>35	28	100.0	3354.70	2729.06
CA-125 at IDS (kU/l)	≤35	10	35.71	20.43	7.96
	>35	13	46.43	203.63	397.36
PFI (months)	Numerical variable	24	85.71	17.44	12.96
Diagnosis (the origin of HGSOC)	1-ovary	8	28.6	/	/
	2 – fallopian tubes	12	42.9	/	/
	3 – peritoneum	8	28.6	/	/
BRCA	0	20	71.4	/	/
	1	6	21.4	/	/
Bevacizumab	0	23	82.1	/	/
	1	4	14.3	/	/
PARPi	0	6	21.4	/	/
	1	21	75.0	/	/

HIF-1α, Hypoxia-inducible factor 1-alpha; PS, Primary surgery; IDS, Interval debulking surgery; KELIM, CA-125 ELIMination Rate Constant K; CRS, Chemotherapy response score; ESR, Erythrocyte sedimentation rate; CRP, C-reactive protein; CA-125, Cancer antigen 125; PFI, Platinum free interval; HGSOC, High-grade serous ovarian cancer; BRCA, BReast CAncer gene; PARPi, Poly (ADP-ribose) polymerase inhibitors.

### 3.2 HIF-1α mRNA expression in different tissue

**Figure 1:**
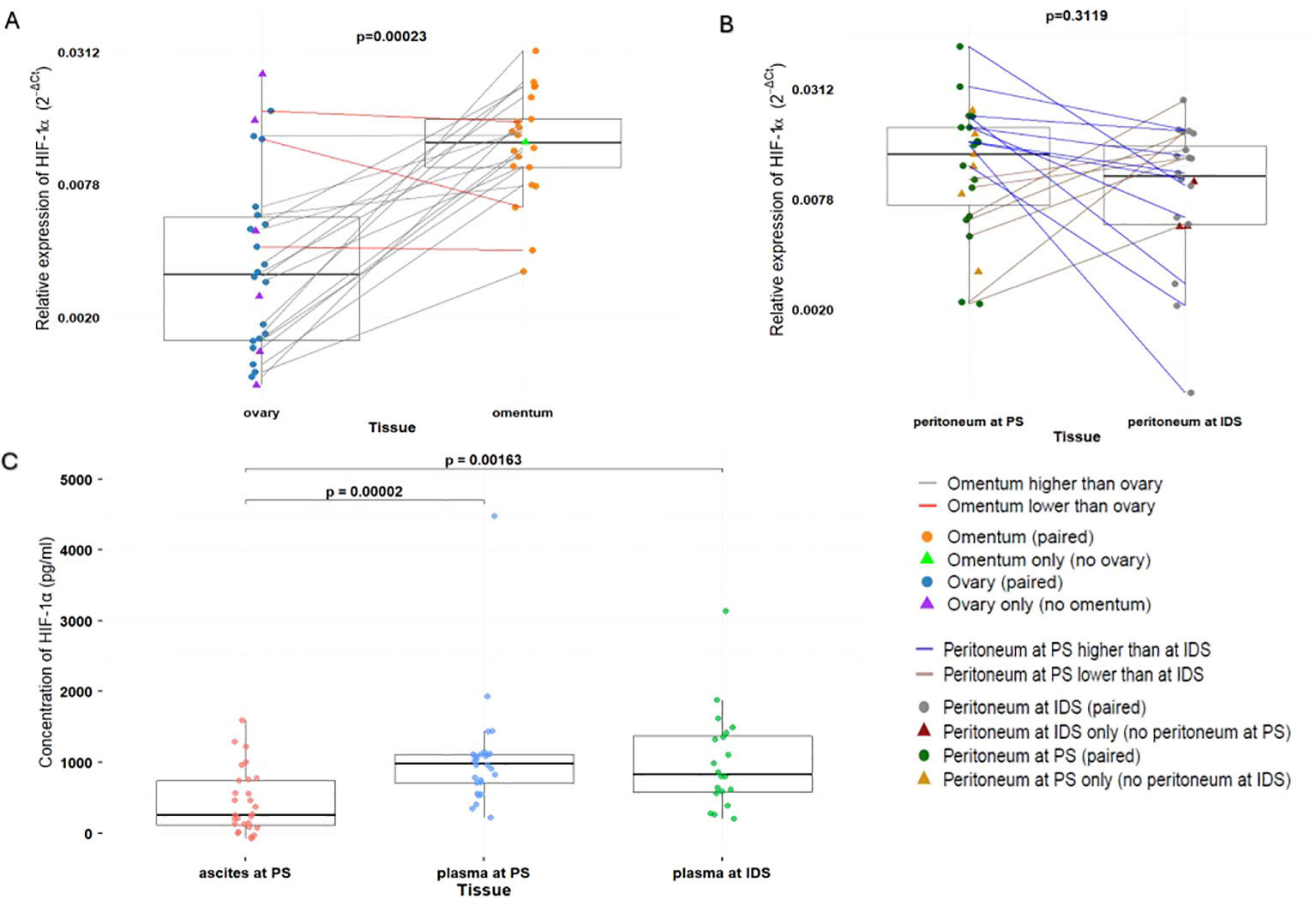
**(A)** mRNA expression of HIF-1α in ovary (n=26) and omentum (n=21). **(B)** mRNA expression of HIF-1α in peritoneum at PS (n=23) and IDS (n=20). **(C)** Concentration of HIF-1α in ascites at PS (n=28), plasma at PS (n=28) and plasma at IDS (n=20). HGSOC, High-grade serous ovarian cancer; ACT, adjuvant chemotherapy; NACT, neoadjuvant chemotherapy; PS, primary surgery; IDS, interval debulking surgery; HIF-1α, Hypoxia-inducible factor 1-alpha.

We analyzed HIF-1α mRNA expression in various tissues at different time points, at PS and at IDS. HIF-1α mRNA expression differed by anatomical site: omental tissue exhibited significantly higher mean mRNA expression compared with ovarian tissue (0.0134 ± 0.007 vs 0.0055 ± 0.006; p = 0.000233), with 85% patients showing higher HIF-1α levels in omentum (**Figure 1A**). In contrast, peritoneal tissue showed no significant change in HIF-1α mean mRNA expression between PS and IDS (0.0151 ± 0.0114 vs 0.0113 ± 0.007; p = 0.3119). However, HIF-1α mRNA expression was higher at PS in 58,8% of patients and lower in 41,2% compared to IDS (**Figure 1B**). When comparing tissue samples collected at the same time, mean HIF-1α mRNA expression was significantly higher in peritoneal tissue than in ovarian tissue collected at PS (p=0.0047), whereas no significant difference was observed between mean mRNA expression in omental and peritoneal tissues collected at IDS (p=0.2465; data not shown).

### 3.3 HIF-1α concentrations in ascites and plasma

We analyzed HIF-1α concentrations in ascitic fluid and plasma collected at PS, and plasma collected at IDS. Mean HIF-1α concentrations in ascitic fluid at PS (443.92 ± 443.43 pg/ml) were significantly lower than those in plasma at PS (1046.59 ± 763.36 pg/ml; p=2.48×10^-5^) and plasma at IDS (1013.22 ± 694.98 pg/ml; p=0.00184). In contrast, comparison of plasma measurements at PS and IDS showed no significant change following treatment (p = 0.41) (**Figure 1C**). Correlation analyses demonstrated significant positive correlation between HIF-1α levels in ascites and plasma at PS (r = 0.631, p = 0.000321; **Supplementary Figure 2A**), between ascites at PS and plasma at IDS (r = 0.472, p = 0.0355; **Supplementary Figure 2B**), and between plasma at PS and plasma at IDS (r = 0.678, p = 0.0010; data not shown). The only statistically significant positive correlation between HIF-1α mRNA expression in tissue and HIF-1α concentrations in body fluids was observed between ovary at PS and plasma at IDS (r = 0.538, p = 0.0175; data not shown).

### 3.4 Link between HIF-1α and systemic inflammation, *BRCA* mutation status and response to chemotherapy

**Figure 2:**
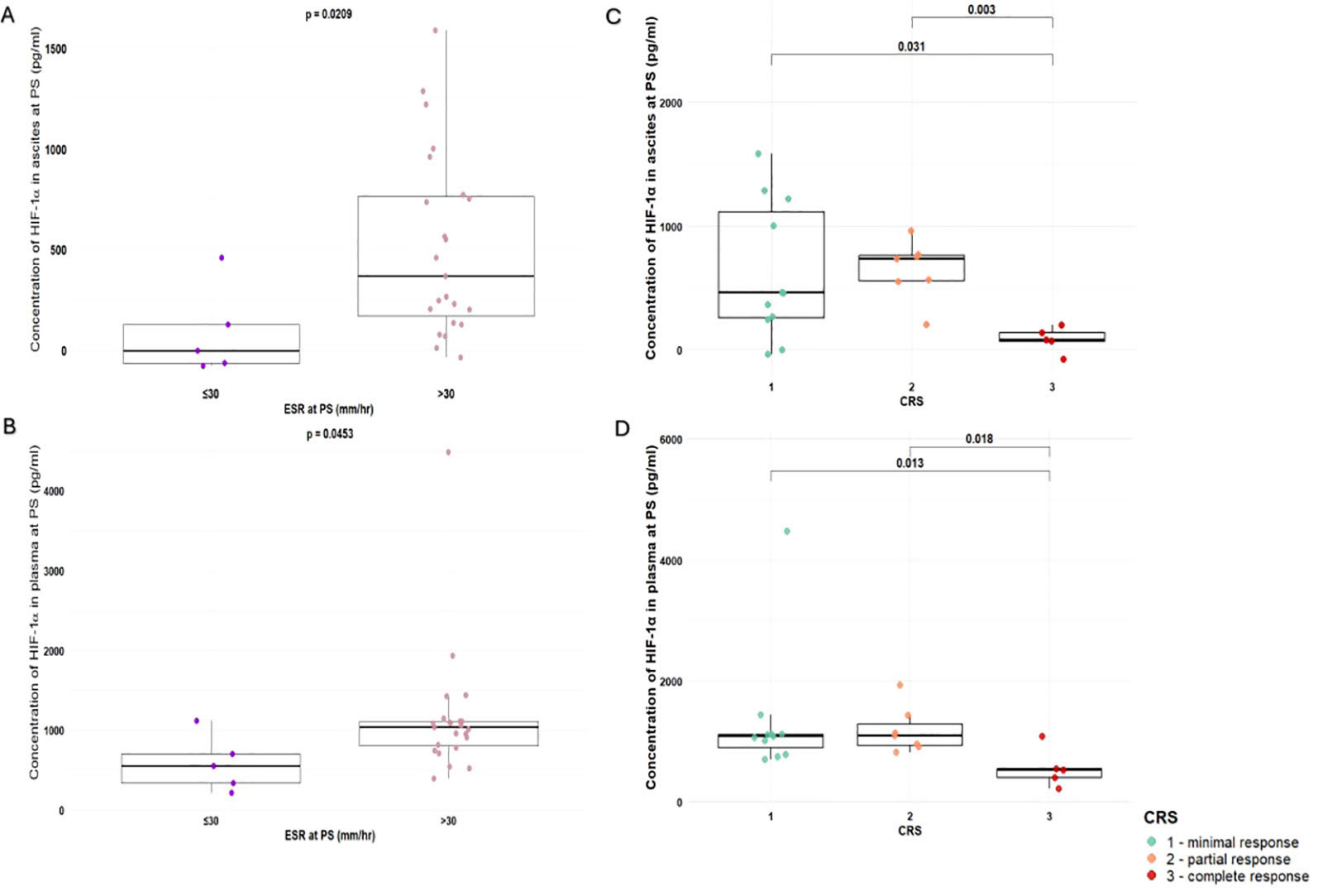
**(A)** Concentration of HIF-1α in ascites at PS grouped by ESR (ESR ≤30 mm/h, n=5; ESR >30 mm/h, n=23). **(B)** Concentration of HIF-1α in plasma at PS grouped by ESR (ESR ≤30 mm/h, n=5; ESR >30 mm/h, n=23). **(C)** Concentration of HIF-1α in ascites at PS grouped by CRS (CRS 1, n=11; CRS 2, n=7; CRS 3, n=5). **(D)** Concentration of HIF-1α in plasma at PS grouped by CRS (CRS 1, n=11; CRS 2, n=7; CRS 3, n=5). PS, primary surgery; IDS, interval debulking surgery; ESR, erythrocyte sedimentation rate; CRS, chemotherapy response score; HIF-1α, Hypoxia-inducible factor 1-alpha.

Mean ESR, a standard laboratory marker for systemic inflammation, was elevated at PS (54.67 ± 25.91 mm/h) and remained elevated at IDS (49.91 ± 27.43 mm/h). An elevated ESR persisted in 72% of patients. We found that patients with an ESR >30 mm/h showed significantly higher HIF-1α levels than those with ESR ≤30 mm/h, in both ascites (514.87 ± 452.34 vs. 117.57 ± 199.6; p = 0.0224) (**Figure 2A**) and plasma at PS (1146.43 ± 796.45 vs. 587.30 ± 351.50; p = 0.0453) (**Figure 2B**). Moreover, at PS, ESR correlated positively with plasma HIF-1α concentration (r = 0.445, p = 0.0175), with CRP (r = 0.447, p = 0.0166), and with *BRCA1/2* (BReast CAncer gene) mutation status (r = 0.431, p = 0.0338).

Similar to ESR, mean CRP level (63.63 ± 54.09 mg/l) was elevated at PS, but unlike ESR, mean CRP level significantly decreased at IDS (10.39 ± 18.39 mg/l, p= 2.1×10^-5^). Only 7% of patients had elevated CRP at IDS. At PS, CRP correlated positively with *BRCA1/2* mutation status (r = 0.438, p = 0.0287). However, CRP and HIF-1α were not significantly linked.

Regarding HIF-1α concentrations in relation to CRS, significant differences were observed between CRS groups in both ascites and plasma at PS. In ascites, mean HIF-1α concentrations differed significantly between CRS1 and CRS3 (627.62 ± 551.51 pg/ml vs. 96.92 ± 75.92 pg/ml; p = 0.041), as well as between CRS2 (649.45 ± 239.80 pg/ml) and CRS3 (p = 0.003) (**Figure 2C**). Similarly, in plasma at PS, significant differences in HIF-1α concentrations were demonstrated between CRS1 and CRS3 (1331.59 ± 1064.40 pg/ml vs. 554.87 ± 324.29 pg/ml; p = 0.013) and between CRS2 (1185.03 ± 384.02 pg/ml) and CRS3 (p = 0.018) (**Figure 2D**). In addition, significant correlation was observed between the concentration of HIF1α in ascitic fluid at PS and CRS (*r* = –0.445, *p* = 0.0434). Importantly, a significant positive correlation was found between CRS and KELIM (*r* = 0.438, *p* = 0.0364). No significant differences in HIF-1α mRNA expression across tissue or fluid compartments, or in ESR or CRP levels, were observed between patients treated with PARP inhibitors or bevacizumab and those who were not.

### 3.5 Association of HIF-1α with PFI and PFS

**Figure 3:**
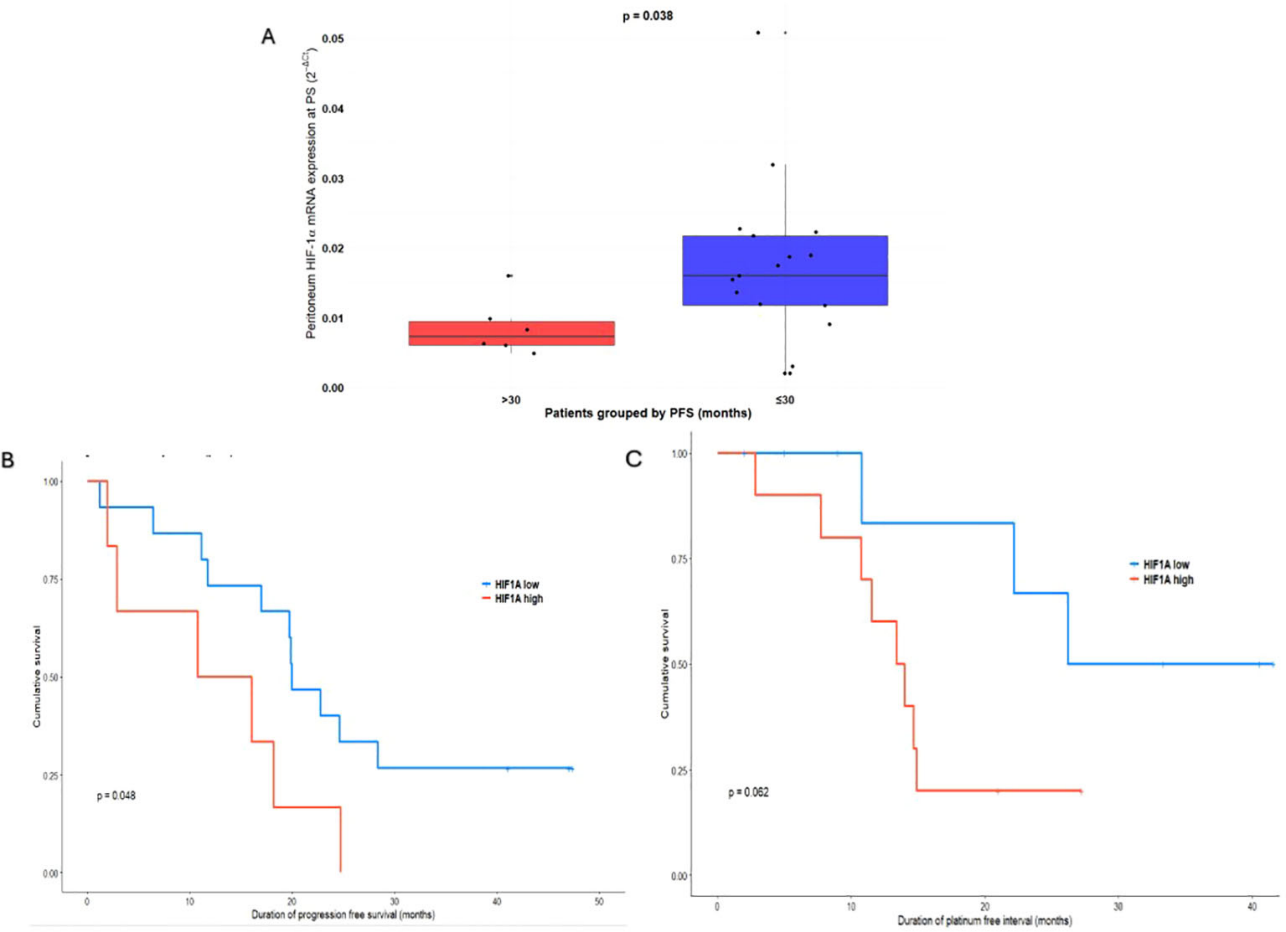
**(A)** mRNA expression of HIF-1α in peritoneum at PS stratified by PFS (PFS ≤30, n=17; PFS >30, n=6). **(B)** Kaplan-Meier survival curve of PFS according to peritoneal HIF-1α mRNA expression. HIF-1α low: peritoneal HIF-1α mRNA expression at PS is below optimal cutoff of 0.01124, n=17; HIF-1α high: peritoneal HIF-1α mRNA expression at PS is above optimal cutoff of 0.01124, n=6. **(C)** Kaplan-Meier survival curve of PFI according to peritoneal HIF-1α mRNA expression. HIF-1α low: peritoneal HIF-1α mRNA expression at PS is below median of 0.01124, n=9; HIF-1α high: peritoneal HIF-1α mRNA expression at PS is above median of 0.01124, n=11. PFS, progression free survival; PFI, platinum free interval; PS, primary surgery; HIF-1α, Hypoxia-inducible factor 1-alpha.

To assess the prognostic value of HIF-1α mRNA expression, we performed statistical analyses using PFS and PFI as clinical endpoint. The median follow-up time in the cohort was 32.5 months, with a total study duration of 55 months, allowing for reliable detection of early and mid-term disease events. The PFS threshold of 30 months was selected based on two factors: (i) the distribution of PFS values in the cohort, where most of the events occurred within the first 30 months, making this threshold a meaningful separator of early, high-risk progression from later, lower-risk recurrence; and (ii) the alignment of this cutoff with the study’s overall 55-month follow-up window. The 30-month PFS threshold should be interpreted as a cohort-specific stratification parameter rather than a clinically validated prognostic cutoff. Patients were stratified by PFS (≤30 vs. >30), revealing a statistically significant difference in HIF-1α mRNA expression in the peritoneum at PS (p = 0.038) (**Figure 3A**). ROC analysis of peritoneal HIF-1α mRNA expression at PS further supported the 30-month PFS threshold, identifying an optimal cutoff of 0.01124, with an AUC of 0.4412. Importantly, this optimal cutoff value closely approximated the mean peritoneal HIF-1α mRNA expression at PS (0.0151 ± 0.0114), indicating that the chosen threshold was biologically grounded rather than artificially imposed. Kaplan–Meier curves for PFS comparing mRNA expression of HIF-1α in peritoneum at PS, divided into two groups (above or below the optimal cutoff), showed a clear separation between groups (p=0.048, **Figure 3B**). In Cox regression, higher peritoneal HIF-1α mRNA expression was associated with an increased risk of early progression (HR = 2.73, 95% CI 0.97–7.67, p = 0.0569), suggesting a clinically meaningful trend.

Kaplan–Meier curves for PFI, stratified by the median peritoneal HIF-1α value at PS (0.0157), demonstrated a clear separation between the high- and low-mRNA expression groups, although the difference did not reach conventional statistical significance (p = 0.062, **Figure 3C**). ROC analysis identified an AUC of 0.5875. Consistently, Cox regression analysis showed a higher risk of chemoresistance associated with elevated peritoneal HIF-1α mRNA expression at PS, with a HR of 3.56 (95% CI: 0.88–14.41, p = 0.0753). Additionally, significant correlations were observed between plasma HIF-1α concentration at PS and PFI (r = –0.440, p = 0.0358). Importantly, PARP inhibitors and bevacizumab used as maintenance therapy did not significantly affect PFS or PFI, nor did they modify the association between HIF-1α expression and these outcomes. KELIM is also significantly corelated with PFI (r = 0.522, p = 0.0126).

## 4 Discussion

Chronic inflammation and hypoxia are well-established contributors to tumor progression, immune dysregulation, and therapeutic resistance in diverse malignancies (1, 2, 5, 17). In HGSOC, however, the spatial heterogeneity of hypoxic signaling and its relationship to systemic inflammation and chemotherapy response remain poorly defined. Previous studies have not evaluated how HIF-1α varies across distinct tumor sites and body compartments, nor whether its dynamics shift following NACT. Our study addresses these questions by integrating multi-compartmental HIF-1α measurements—tumor tissue, ascitic fluid, and plasma from the same patient—at baseline and after chemotherapy, and by linking these findings with standard clinical inflammatory markers, chemotherapy response indicators and disease progression. This approach provides a more comprehensive understanding of hypoxia–inflammation interactions in HGSOC than previously available (18–20).

The analysis of HIF-1α mRNA expression showed, compared to ovarian tissue at baseline, higher mRNA expression in peritoneal tissue at baseline, as well as increased mRNA expression in both the omentum and peritoneal tissue after NACT. Additionally, the average mRNA expression of HIF-1α in the peritoneum before and after NACT did not show a significant difference. The consistently higher HIF-1α mRNA expression in the omentum compared with the ovary aligns with the biological specialization of the omentum, whose macrophage- and lymphocyte-rich milky spots create a microenvironment predisposed to persistent inflammatory activation. These structures generate cytokines and reactive oxygen species (ROS)—including TNF-α and IL-1β—that can stabilize HIF-1α and promote a hypoxia–inflammation feedback loop. While our findings do not establish mechanism, the elevated omental HIF-1α aligns with previous descriptions of the omentum as a pro-inflammatory, tumor-supportive niche (6, 17, 21, 22). Importantly, peritoneal HIF-1α remained largely unchanged following NACT, despite significant reductions in tumor volume typically observed in HGSOC. This stability implies that hypoxic signaling in the peritoneal microenvironment may be relatively resistant to chemotherapy-induced modulation and may support the persistence of residual tumor cells and subsequent disease recurrence. Prior studies have focused on bulk responses without site-specific analysis of hypoxic markers (18); thus, the identification of a persistent peritoneal HIF-1α signature represents a novel contribution.

Our findings also reveal distinct but interconnected HIF-1α profiles in ascites and plasma. At both PS and IDS, HIF-1α concentrations were lower in ascites than in paired plasma. This difference may reflect biological or biochemical factors, including limited secretion of soluble HIF-1α into the peritoneal fluid or increased proteolytic degradation within the ascites compared to plasma, which is known to contain high levels of proteases and fragmented proteins (23). Another important finding is that mean plasma HIF-1α concentration remained elevated after NACT, suggesting persistent systemic hypoxia-related signaling. Despite the quantitative differences between compartments, significant correlations were present between ascitic and plasma HIF-1α at both PS and IDS, and between tissue and fluid measures such as ovarian HIF-1α at PS and plasma at IDS. These cross-compartment correlations suggest a potential link between local hypoxic conditions within the tumor microenvironment and systemic hypoxic or inflammatory responses. This finding supports a model in which hypoxia-driven signaling operates across both localized and systemic levels (9). Moreover, positive correlation between ovarian HIF-1α mRNA expression at PS and plasma concentrations at IDS may reflect an intrinsically hypoxia-driven tumor phenotype in which hypoxia-related pathways may remain active regardless of chemotherapy response. However, given the exploratory design, these associations should be interpreted cautiously.

The systemic inflammatory marker ESR showed a significant associations with HIF-1α in this cohort. An ESR ≥30 mm/h, a clinical threshold indicating chronic inflammatory activation (24), was associated with higher HIF-1α concentrations in both ascites and plasma at PS. Notably, ESR levels remained elevated following NACT, whereas CRP levels decreased to normal in most patients and were not associated with HIF-1α concentration in body fluids. This divergence may reflect difference in the biological properties of these markers: ESR captures sustained, chronic inflammatory activity, whereas CRP is a rapidly responding acute-phase reactant that typically normalizes following cytotoxic treatment (25–27). In this context, Hypoxia-driven signaling mediated by HIF-1α may be more closely associated with persistent inflammatory activation than with transient inflammatory responses, potentially explaining the lack of association with CRP (17, 28). To our knowledge, an association between ESR and HIF-1α has not been previously reported in ovarian cancer studies. The observed relationship suggests a potential link between systemic inflammation and hypoxia-related signaling, although whether elevated ESR contributes to HIF-1α stabilization or represents a downstream consequence of hypoxia-driven pathway cannot be determined from this study (28–30).

The relationship between BRCA1/2 status and inflammation has also gained attention in recent years. BRCA1 has been shown to influence the hypoxic response and HIF-1α stability, and BRCA1-mutant cancers display a HIF-1α–high phenotype in several immunohistochemical studies. BRCA1/2 deficiency activates the cGAS–STING pathway, enhancing cytokine production including IL-6—the principal driver of CRP (31, 32). Although direct clinical evidence linking BRCA1/2 mutations to systemic inflammatory markers remains limited, our observation that ESR and CRP correlated with BRCA mutation status aligns with these emerging mechanistic models and should be viewed as hypothesis-generating.

Next, we observed that elevated HIF-1α concentrations in ascites and plasma at PS—not in omental tissue—were associated with lower CRS, indicating reduced responsiveness to platinum-based chemotherapy. This result was unexpected since the omentum is the recommended and validated site for CRS assessment (33). However, ascites and plasma may reflect a more systemic or fluid environment, possibly representing overall tumor burden or hypoxic signaling released or shed by tumor cells throughout the body. In this context, our observation is consistent with evidence from other malignancies in which hypoxia and elevated HIF-1α contribute to metabolic reprogramming, enhanced DNA repair, and inhibition of apoptosis, all of which reduce chemotherapy effectiveness (34, 35). Additionally, we observed the expected positive correlation between KELIM and CRS, consistent with prior studies reporting that KELIM <1 is associated with lower CRS (14, 36).

Predicting recurrence in HGSOC is critical for personalized patient management and improving prognosis, particularly given the generally poor long-term survival associated with this disease (37). In this context, exploratory analyses suggested that stratification by 30-month PFS was associated with higher peritoneal HIF-1α mRNA expression at PS in patients experiencing early recurrence. Kaplan–Meier analysis indicated shorter PFS in patients with higher peritoneal HIF-1α mRNA expression. The univariate Cox regression analysis suggested a trend toward increased risk, although the estimate should be interpreted with caution given the borderline statistical significance. These observations are consistent with previous findings by Chen et al. who reported that high HIF-1α mRNA expression in epithelial ovarian cancer was associated with shorter PFS (19). Because PFI is a key indicator of response to subsequent chemotherapy at recurrence, we further explored its relationship with HIF-1α. Stratification by the median peritoneal HIF-1α value at PS suggested shorter PFI in patients with higher mRNA expression. Kaplan–Meier analysis demonstrated a clear separation between groups, though the difference did not reach statistical significance and Cox regression suggested a more than threefold increased risk of chemoresistance. In line with this exploratory pattern, plasma HIF-1α concentration at PS showed a negative correlation with PFI, whereas KELIM s was positively correlated with PFI. Our result is in line with the findings of Daponte et al., who reported that high HIF-1α mRNA expression was associated with shorter PFI (20). Collectively, these results suggest that peritoneal HIF-1α may be associated with disease progression and treatment resistance and could represent a candidate marker for further investigation in HGSOC. However, this observation should be interpreted with caution and requires independent validation before any prognostic or predictive implications can be drawn.

## 5 Limitations and strengths of our study

This study has several limitations that should be acknowledged. First, the retrospective, single-center design and the relatively small sample size limit the generalizability of the findings and reduce statistical power. Second, the limited cohort size precluded the inclusion of comprehensive multivariable analyses, and the results should therefore be interpreted with appropriate caution. Specifically, we were unable to adjust for potential confounders such as FIGO stage, BRCA/HRD status, treatment regimen variations, and residual disease after surgery. The potential impact of PRAP inhibitors and bevacizumab was explored in secondary analyses; however, as these agents were introduced after IDS, they could not have influenced the CRS which was assessed at IDS. Their potential impact on OS and PFS was examined separately. In addition, although biomarker selection was hypothesis-driven and based on biological rationale, these analyses were exploratory in nature and intended to generate hypotheses rather than establish definitive conclusions. Given these considerations, independent validation in larger, similarly defined cohorts will be essential to confirm the robustness and broader applicability of the observed findings. Further functional and mechanistic studies are needed to elucidate the role of HIF-1α in inflammation-associated chemoresistance, and protein-level assessments of HIF-1α should complement mRNA analysis.

Despite these limitations, the study benefits from its integrative, multi-compartment design, incorporating HIF-1α measurements from tumor tissue, ascites, and plasma at both PS and IDS, enabling a comprehensive assessment of hypoxic signaling across local and systemic environments. Combining molecular data with inflammatory markers, chemotherapy response metrics, and survival outcomes provides a multidimensional framework not previously explored in HGSOC. Paired sampling further offers insight into treatment-resistant hypoxic niches persisting despite NACT. Although the single-center design restricts the number of patients, it allows for consistent treatment protocols and rigorous standards for sample preparation and quality.

## 6 Conclusion

This study shows that hypoxia-driven inflammatory signaling, reflected by HIF-1α across tumor tissues, ascites, and plasma, is a persistent biological feature of HGSOC. Elevated HIF-1α mRNA expression in the omentum and peritoneum highlights these sites as key immunometabolic niches that sustain chronic inflammation. Notably, peritoneal HIF-1α levels remained unchanged after treatment, highlighting that current chemotherapy fails to diminish the hypoxic–inflammatory environment. Because this stable niche may contribute to chemoresistance and poorer outcomes, it warrants further investigation as potential therapeutic target in larger independent cohorts before clinical translation can be considered. Future studies should evaluate strategies that directly modulate this peritoneal hypoxic–inflammatory milieu.

## Data Availability

The raw data supporting the conclusions of this article will be made available by the authors, without undue reservation.
