# Supplementary material for: Integrating chronic inflammation and hypoxia: the potential role of HIF-1α in tumor behavior and therapy response in high-grade serous ovarian cancer
Source: Front Immunol. 2026 Mar 9;17:1757708. doi: 10.3389/fimmu.2026.1757708 (PMC13006265; doi:10.3389/fimmu.2026.1757708)
Supplement: Supplementary file 1 [file DataSheet1.docx]

Supplementary Material

# Study design

Our study was a retrospective clinical trial involving patients diagnosed with advanced-stage (FIGO stage III or IV) primary high-grade serous ovarian cancer (HGSOC). The inclusion criteria comprised: (i) a confirmed diagnosis of primary HGSOC at FIGO stage III or IV; (ii) presence of ascites: (iii) absence of concurrent malignant neoplasms; and (iv) referral for neoadjuvant chemotherapy (NACT). A total of 45 women with suspected ovarian cancer were initially enrolled and referred for laparoscopy or laparotomy, depending on disease extent, overall performance status, and findings from gynecological examination and computed tomography. Among them, 28 were referred to NACT after diagnostic laparoscopy or exploratory laparotomy, which were considered part of the primary surgical procedure (PS) (Supplementary Figure 1.). Among 28 referred for NACT, 5 did not complete the planned treatment protocol. As part of the NACT regimen, individuals were administered 3–6 cycles of platinum-based medicines (Pt-BMs) prior to interval debulking surgery (IDS) and an additional 0–3 cycles postoperatively. Standard ACT/NACT regime for HGSOC consisted of carboplatin combined with paclitaxel. In cases where a patient’s clinical condition did not allow combination therapy, carboplatin monotherapy was used. Maintenance therapy with Poly (ADP-ribose) polymerase (PARP) inhibitors (niraparib or olaparib) was introduced afterward when indicated. Bevacizumab, an anti-VEGF monoclonal antibody, was incorporated into the chemotherapy regimen when clinically appropriate. For each patient, blood, ascites, and ovarian and peritoneal tissue samples were collected at PS, while blood, peritoneal, and omental tissue samples were obtained at. Ascites was not collected at IDS due to its absence in many patients following successful NACT. The omentum was selected for analysis instead of the ovary because it is a common site of metastatic disease and provides a more representative assessment of tumor response to NACT. To evaluate tumor response to NACT we used CRS, KELIM and PFI. CRS is a validated histopathological system that measures the extent of tumor regression by examining alterations in tumor cells and the surrounding stroma in omentum. The system then categorizes the response into three tiers: CRS1 (no or minimal response), CRS2 (partial response), and CRS3 (complete or near complete response). KELIM is a mathematical model that describes the rate of decline of CA125 as a dynamic indicator of intrinsic tumor chemosensitivity. Higher KELIM values reflect faster CA125 elimination and correlate with a more favorable response to platinum-based chemotherapy and improved long-term outcomes. The official cutoff for KELIM was used: values <1.0 were considered unfavorable, whereas values ≥1.0 were considered favorable. KELIM was calculated with at least three observed CA-125 values (prior to cycle 2, 3 and 4) during the first 100 days of NACT.PFI was calculated as the time interval between the last cycle of platinum-based chemotherapy and the initiation of a new chemotherapy regimen at disease recurrence or the occurrence of death, whichever came first. PFI is crucial in ovarian cancer as it helps to determine the effectiveness of retreatment. A longer interval suggests a higher likelihood of response to subsequent platinum-based chemotherapy

# Supplementary Figures


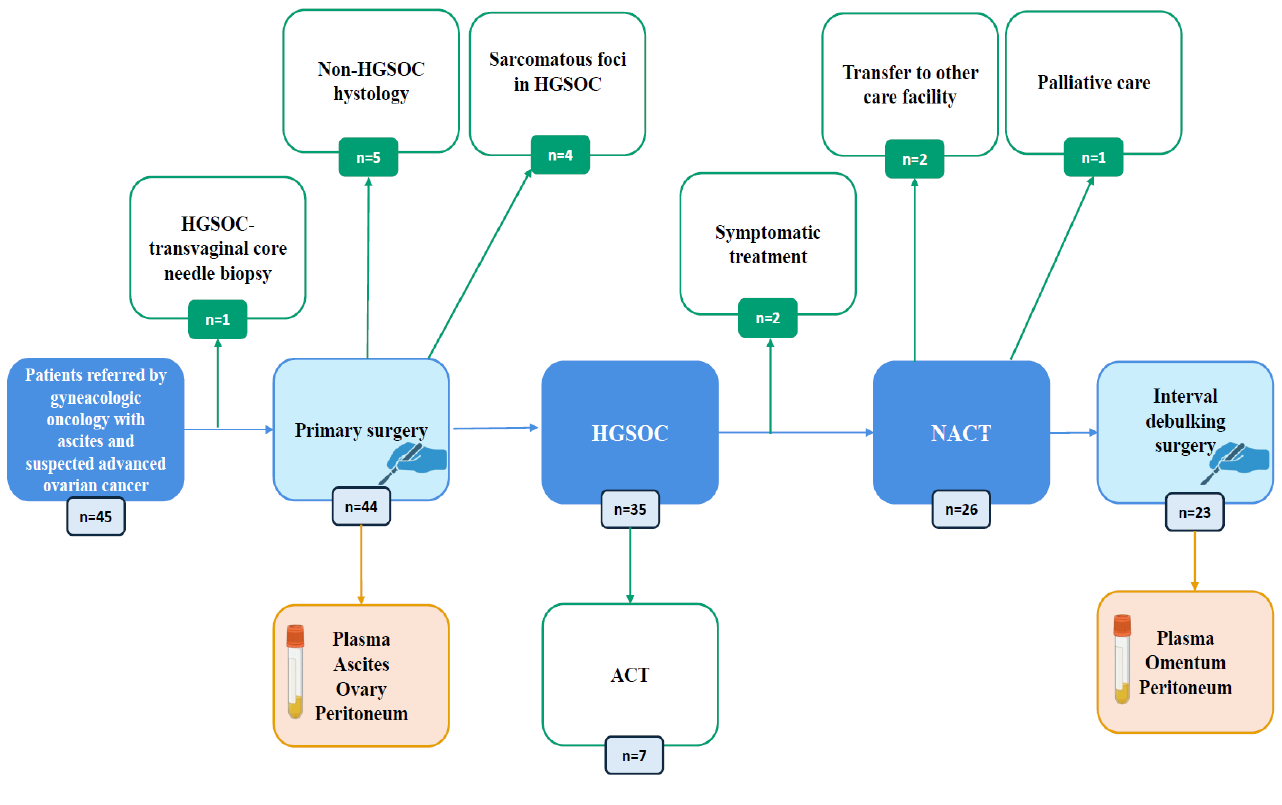


**Supplementary Figure 1.** Study protocol and sample collection timeline and patient dropout reasons through the study. HGSOC - High-grade serous ovarian cancer, ACT - adjuvant chemotherapy, NACT - neoadjuvant chemotherapy.


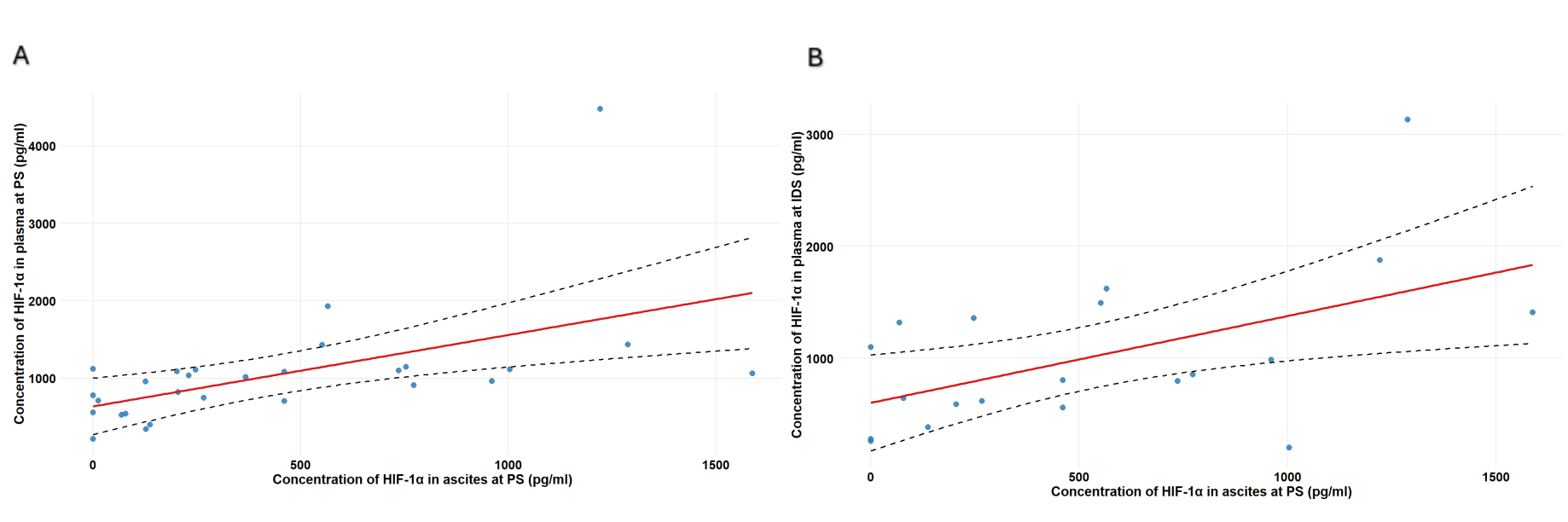


**Supplementary Figure 2.** (A) Correlation between concentration of HIF-1α in ascites at PS and plasma at PS (n=28). (B) Correlation between concentration of HIF-1α in ascites at PS and plasma at IDS (n=20). PS - primary surgery, IDS - interval debulking surgery, HIF-1α – Hypoxia-inducible factor 1-alpha.
